# Dynamics of fungal communities during *Gastrodia elata* growth

**DOI:** 10.1186/s12866-019-1501-z

**Published:** 2019-07-10

**Authors:** Lin Chen, Yu-Chuan Wang, Li-Yuan Qin, Hai-Yan He, Xian-Lun Yu, Ming-Zhi Yang, Han-Bo Zhang

**Affiliations:** 1 State Key Laboratory for Conservation and Utilization of Bio-Resources in Yunnan, Yunnan University, Kunming, 650091 China; 2 School of Ecology and Environmental Science, Yunnan University, Kunming, 650091 China; 3 School of Life Sciences, Yunnan University, Kunming, 650091 China; 4 Gastrodia Tuber Research Institute of Zhaotong, Zhaotong, 657000 Yunnan Province China

**Keywords:** *Gastrodia elata*, Growth phases, *Mycena*, High-throughput sequencing, Fungal dynamics

## Abstract

**Background:**

*Gastrodia elata* is a widely distributed achlorophyllous orchid and is highly valued as both medicine and food. *Gastrodia elata* produces dust-like seeds and relies on mycorrhizal fungi for its germination and growth. In its life cycle, *G. elata* is considered to switch from a specific single-fungus relationship (*Mycena*) to another single-fungus relationship (*Armillaria*). However, no studies have investigated the changes in the plant-fungus relationship during the growth of *G. elata* in the wild. In this study, high-throughput sequencing was used to characterize the fungal community of tubers in different growth phases as well as the soils surrounding *G. elata*.

**Results:**

The predominant fungi were Basidiomycota (60.44%) and Ascomycota (26.40%), which exhibited changes in abundance and diversity with the growth phases of *G. elata*. Diverse basidiomycetes in protocorms (phase P) were *Hyphodontia*, *Sistotrema*, *Tricholoma*, *Mingxiaea*, *Russula*, and *Mycena*, but the community changed from a large proportion of *Resinicium bicolor* (40%) in rice-like tubers (phase M) to an unidentified Agaricales operational taxonomic unit 1(OTU1,98.45%) in propagation vegetation tubers (phase B). The soil fungi primarily included *Simocybe*, *Psathyrella*, *Conocybe*, and *Subulicystidium*. Three *Mycena* OTUs obtained in this study were differentially distributed among the growth phases of *G. elata*, accounting for less than 1.0% of the total reads, and were phylogenetically close to *Mycena epipterygia* and *M. alexandri*.

**Conclusions:**

Our data indicated that *G. elata* interacts with a broad range of fungi beyond the *Mycena* genus. These fungi changed with the growth phases of *G. elata*. In addition, these data suggested that the development of the fungal community during the growth of *G. elata* was more complex than previously assumed and that at least two different fungi could be involved in development before the arrival of *Armillaria*.

**Electronic supplementary material:**

The online version of this article (10.1186/s12866-019-1501-z) contains supplementary material, which is available to authorized users.

## Background

Orchidaceae has been considered to be the most diverse and widely distributed plant family on earth [1]. Because orchids produce tiny, dust-like seeds [2], which lack the necessary energy reserves, a variety of mycorrhizal fungi are required to stimulate their germination and seedling growth, a strategy termed mycoheterotrophy [3, 4]. All orchids form protocorms at the beginning of germination [4, 5]. In the basal region of the protocorm, most plant cells are observed to harbor viable, coiled intracellular fungal hyphae (the *pelotons*) that eventually collapse and undergo lysis. After this early phase of seedling, most orchids develop green leaves and photosynthesize and are known as partial mycoheterotrophs. However, there are also > 200 achlorophyllous orchid species, which obtain their complete nutrients and carbon supply from mycorrhizal fungi and are known as full mycoheterotrophs [1, 6].

Because orchids are partially or fully dependent on mycorrhizal fungi, the mycorrhizal communities associated with orchids could be expected to mediate the abundance, spatial distribution and coexistence of terrestrial orchids in natural communities [7]. Some species of orchids have a narrow taxonomic group of mycorrhizal fungi [8]. For example, the fully mycoheterotrophic orchid *Corallorhiza maculata* is colonized exclusively by the same highly specific ectomycorrhizal fungi throughout the year [9]. Shefferson et al. (2008) found that the mycorrhizal fungi associated with orchids that colonized mine tailing hills were the same as those in pristine habitats [10]. Jacquemyn et al. (2014) indicated that cooccurring orchid species have distinctive mycorrhizal communities and show strong spatial segregation [11]. However, the easy germination and protocorm formation of most orchids in unoccupied habitats strongly suggest the ubiquitous occurrence and low specialization of mycorrhizal fungi [12–14]. For example, species in the genus *Dactylorhiza* have been associated with a diverse suite of mycorrhizal fungi [15]. Tĕšitelová et al. (2012) found evidence for low fungal specificity in four *Epipactis* species with divergent ecological preferences [16]. In addition, *Cynorkis purpurea* was found to be a generalist with diverse mycorrhizal fungi spanning 12 operational taxonomic units (OTUs) in three genera (*Tulasnella*, *Ceratobasidium*, and *Sebacina*) [17].

Interestingly, throughout their life history, the fungal specificity patterns in orchid seedlings and adults vary among orchid species [18]. The same fungi facilitate both seed germination and nutrition of the adult plants of *Drakaea* spp. [19]. However, other species switch from a single-fungus relationship to relationships with a variety of fungi, e.g., *Tipularia discolor* [20] and *Orchis purpurea* [21]. In addition, seedlings of *Cephalanthera* or *Epipactis* associate with a selection of fungi and continue with a wider selection in adulthood [16, 22].

*Gastrodia elata* is an achlorophyllous orchid and is primarily distributed in East Asia, Southeast Asia and Oceania [23, 24]. Its rhizome (mature tuber) is a popular herbal medicine in Asian countries, such as Korea, China and Japan [25, 26]. The rhizome has also been used as a food and is commonly cooked in soups. Like many other orchids, *G. elata* produces thousands of dust-like seeds (10,000–50,000 seeds per capsule) [27]. These seeds do not possess an endosperm and thus have to germinate and develop into protocorms, forming vegetative propagation corms and immature tubers only when adequate nutrients are obtained through the digestion of the specific fungi, *Mycena* spp. *G. elata* subsequently undergoes vegetative growth through an established symbiotic association with the compatible mycorrhizal fungus *Armillaria mellea* to yield mature tubers [28, 29]. Therefore, *G. elata* is widely considered to switch from a specific single-fungus relationship (*Mycena*) to another single-fungus relationship (*Armillaria*) from germination to adult growth [30]. Some species of *Gastrodia*, for example, the adult *G. confusa,* also gain carbon through a narrow taxonomic group of *Mycena* species [31].

However, other *Gastrodia* species have been shown to associate with diverse litter- or wood-decaying fungi, e.g., *Resinicium* in *G. similis* [32] and *Campanella* and *Marasmius* in *G. sesamoides* [33]. Although three closely related species—*G. confusa*, *G. pubilabiata*, and *G. nipponica*—have different fungal specificities, no shift of fungal partners in the course of the life history occurs [34]. There have been no detailed studies on the fungal community associated with *G. elata* in the wild since 1989, when the fungus *Mycena osmundicola* was isolated from *G. elata* protocorms for the first time [35]. Although in vitro studies have shown that several *Mycena* species, including *M. orchidicola*, *M. dendrobii*, and *M. anoectochili*, promote the germination of *G. elata*, these fungi were isolated from the orchids *Cymbidium sinense, Dendrobium candidum*, and *Anoectochilus roxburghii*, respectively [36]. Therefore, a final conclusion has not yet been reached on the fungal switch during the development phases of *G. elata*.

To accurately describe mycorrhizal associations in *G. elata*, fungal communities should be assessed rather than the presence of individual fungal species, as has been previously shown [29]. In addition, more advanced detection techniques are required rather than traditional isolation and culture methods. Novel high-throughput sequencing methods, such as Illumina sequencing and PacBio sequencing, have recently been used to explore the fungal communities associated with orchids, including species from five different genera (*Anacamptis*, *Neotinea*, *Orchis*, *Ophrys* and *Serapias*) in Gargano National Park in southern Italy [11] and *G. flavilabela* in Taiwan [24]. The present study is the first example of the use of high-throughput sequencing to characterize the fungal community associated with *G. elata* in Zhaotong, China. We aimed to understand (1) the dynamics of the fungal community associated with *G. elata* in different developmental phases, (2) whether the *Mycena* species comprise the majority of fungi associated with *G. elata* during early development phases, and (3) the overlap between the fungal communities associated with *G. elata* and those in the surrounding soils.

## Results

### Fungal samples and ITS sequences

From the genomic DNA of each sample, the internal transcribed spacer 1 (ITS1) rDNA segments were amplified, and the PCR products were subjected to Illumina MiSeq sequencing. A total of 402,117 high-quality reads were obtained from 12 samples (including nine plant tuber samples and three methodological replicates of soil, which were performed to increase the recovery of fungi from the pooled soil samples), and 1000 OTUs were observed at a 97% similarity. Each sample contained 4531 to 41,185 reads with different phylogenetic OTUs ranging from 30 to 597 (additional table files provide greater detail [see Additional file 1: Table S1 and Additional file 2: Table S2]). After subsampling, 779 OTUs were observed at 97% similarity, with different OTUs ranging from 14 to 373 (additional files show these findings in greater detail [see Additional file 1: Table S1 and Additional file 3: Table S3]). The phase B samples provided the fewest OTUs and the greatest number of unidentified fungi OTUs. The OTU table after subsampling offers greater detail (see Additional file 3: Table S3).

All the rarefaction curves tended to approach the saturation plateau, suggesting a reasonable number of sequenced reads in each sample. The species accumulation curves showed that these fungal diversity indices declined with the growth of *G. elata* (Additional file 4: Figure S1).

### Fungal communities of the tubers and surrounding soils

**Fig. 1:**
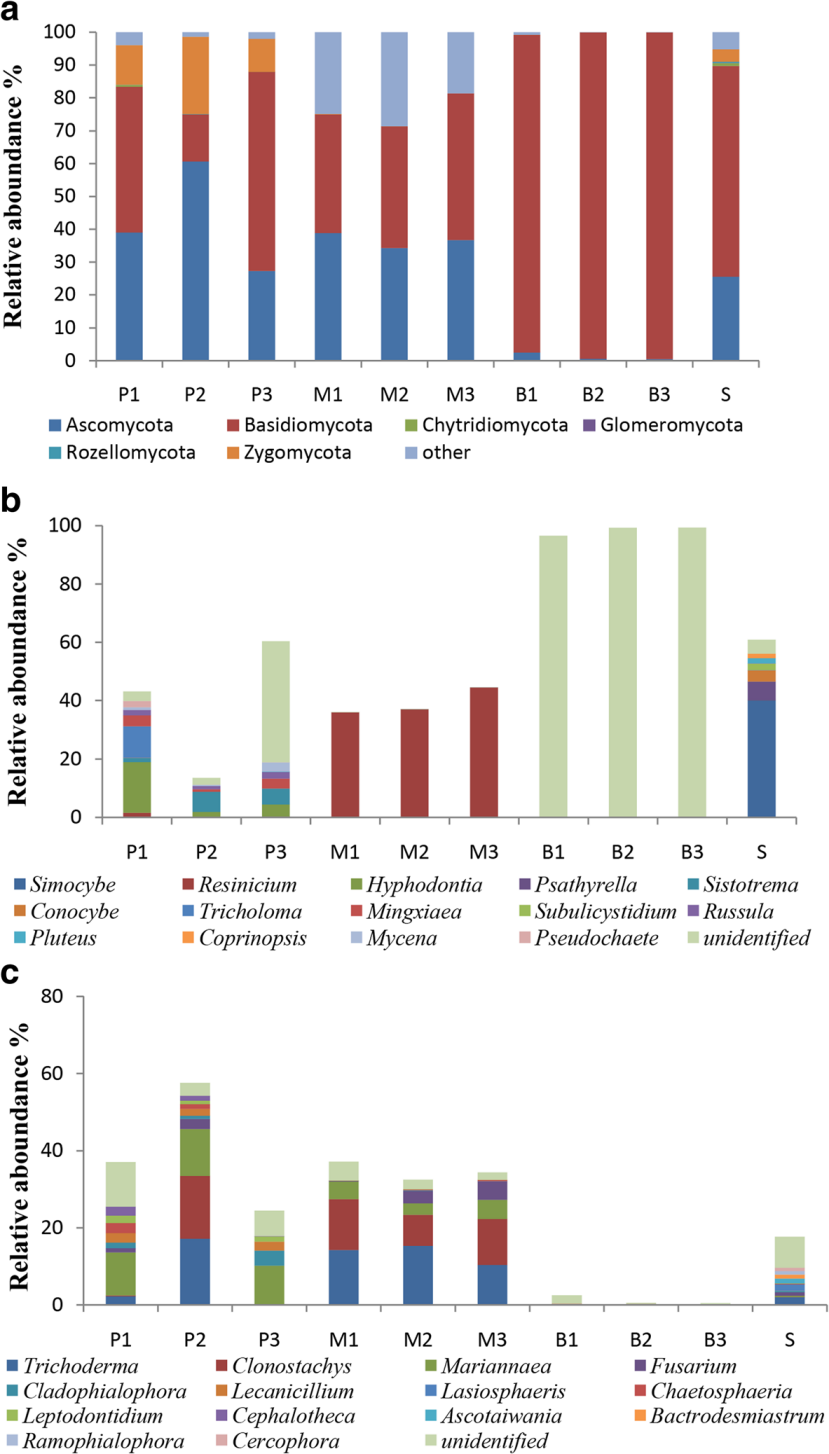
Distribution histogram of fungal community structure. Distribution histogram of fungal community structure at the phylum level (**a**) in Basidiomycota (**b**) and in Ascomycota (**c**). “Other” includes those nonfungal and unidentified sequences at the phylum level. P: protocorms; M: rice-like *G. elata*; B: propagation vegetation tubers; and S: surrounding soils

At the phylum level, the predominant fungi in all the samples, including the tubers and soils, were from Basidiomycota and Ascomycota, with average abundances of 60.44 and 26.40%, respectively (Fig. 1a). The annotated fungi in the protocorm (phase P) belonged to five phyla, with the three most abundant phyla being Ascomycota, Basidiomycota and Zygomycota, accounting for 42.33, 39.76 and 15.21%, respectively. The fungi of rice-like *G. elata* (phase M) were assigned to three phyla. Among them, Basidiomycota and Ascomycota were dominant, accounting for 39.29 and 36.62%, respectively. The fungi of the propagation vegetation tubers (phase B) included three phyla, with 98.48% from Basidiomycota. Basidiomycota and Ascomycota accounted for 60.44 and 26.41%, respectively, in the surrounding soils.

At the genus level, the distribution of Basidiomycota and Ascomycota varied among the growth phases of *G. elata* (Fig. 1b, c). The abundance of Basidiomycota typically increased with the growth phases of *G. elata* (Fig. 1b). Although relatively diverse fungi were found in phase P, including *Hyphodontia* (7.8%), *Sistotrema* (4.58%)*, Tricholoma* (3.58%)*, Mingxiaea* (2.66%)*, Russula* (1.79%)*,* and *Mycena* (1.47%), in total, these genera accounted for nearly 20% of fungi in this phase. *Resinicium* was numerically dominant in phase M, with a percentage of 39.30%. Much less diverse fungi were found in the propagation vegetation tubers (phase B), and more than 98.45% were in Agaricales. Soil fungi belonged to four genera with an average percentage > 2.0%, including *Simocybe* (40.12%), *Psathyrella* (6.35%), *Conocybe* (3.91%), and *Subulicystidium* (2.30%).

In contrast, the abundance of Ascomycota decreased with the growth phases of *G. elata* (Fig. 1c). The fungi in phase P were primarily *Mariannaea* (11.07%)*, Trichoderma* (6.48%)*, Clonostachys* (5.56%)*, Lecanicillium* (2.20%) and *Cladophialophora* (2.06%). However, the majority of fungi in phase M were *Trichoderma* (13.29%) and *Clonostachys* (11.08%), and phase B rarely contained Ascomycota fungi. The soils primarily contained *Trichoderma* (1.96%)*, Lasiosphaeris* (1.70%)*, Ascotaiwania* (1.21%) and *Bactrodesmiastrum* (1.02%).

**Fig. 2:**
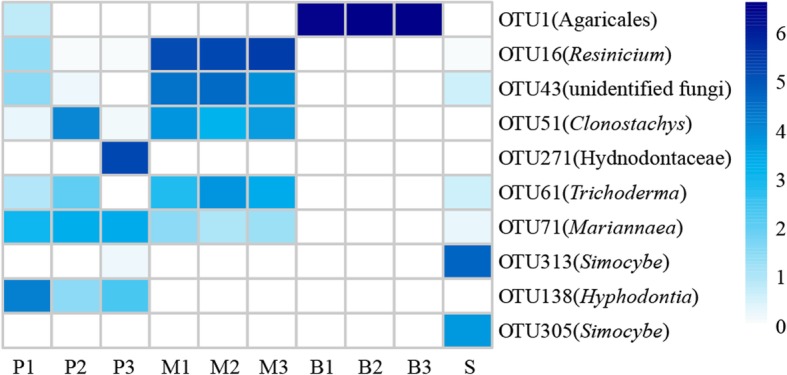
Heatmap of the distribution of the top 10 OTUs among the different groups. The relative abundances are expressed as the richness of fungi transformed by log_2_(x + 1) in a given sample. P: protocorms; M: rice-like *G. elata*; B: propagation vegetation tubers; and S: surrounding soils

**Fig. 3:**
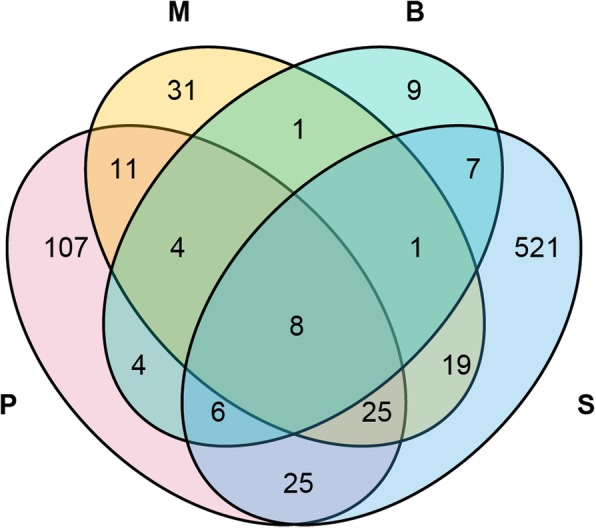
Venn diagram for the four fungal communities. The numbers are the OTU number in each part. P: protocorm; M: rice-like tubers; B: propagation vegetation tubers; S: surrounding soils

Consequently, the distribution of the top 10 OTUs, which accounted for 63.65% of the total fungi detected, including six basidiomycetes, three ascomycetes and one unidentified fungus, were used to compare the difference in the fungal community among groups (Fig. 2, also see Additional file 3: Table S3 for the OTU table after subsampling). The average abundances of OTU71 (*Mariannaea samuelsii*, Ascomycota), OTU138 (*Hyphodontia barba-jovis*, Basidiomycota) and OTU271 (Hydnodontaceae, Basidiomycota) declined with the growth of *G. elata.* OTU71 and OTU271 were primarily found in the protocorms. Two *Simocybe* spp. from Basidiomycota, including OTU313 and OTU305, were almost exclusively detected in soils. An Agaricales OTU1 was the most abundant in phase B. In addition; other OTUs, including OTU16 (*Resinicium bicolor*, Basidiomycota), OTU43 (unidentified fungus), OTU51 (*Clonostachys* sp., Ascomycota) and OTU61 (*Trichoderma harzianum*, Ascomycota), showed an increase in the first two phases of *G. elata* (P and M) and then a decrease in phase B. Totally, the numbers of unique OTUs from different groups were much higher than those of shared OTUs (Fig. 3, also see Additional file 5: Table S4).

### Phylogenetics and distribution of *Mycena* sp. during *G. elata* germination

**Fig. 4:**
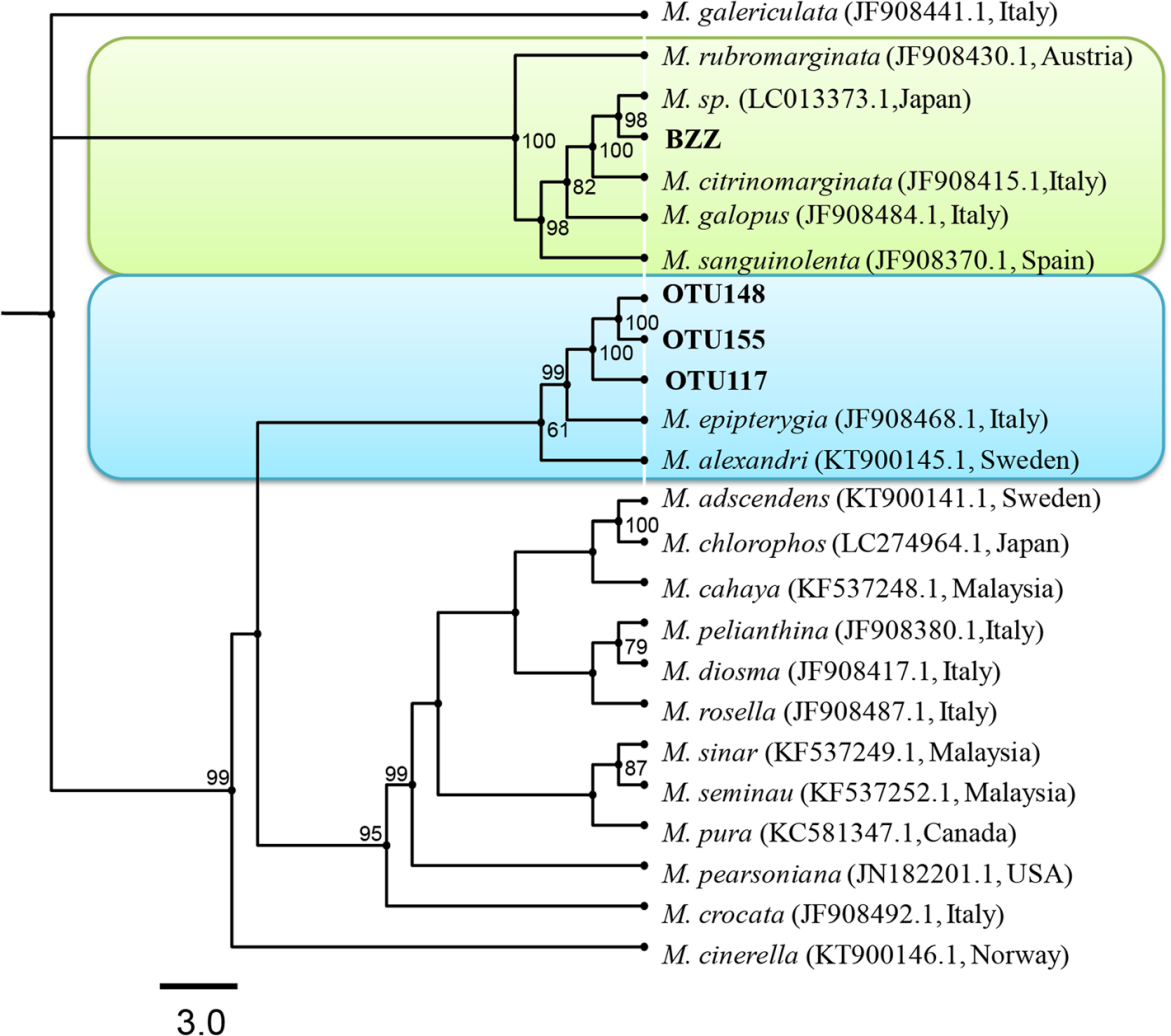
Phylogenetic tree of *Mycena*spp. Three *Mycena* detected in this study were designated OTU117, OTU155 and OTU117. The strain BZZ was previously isolated from a protocorm of *G. elata*. Bootstrap values of > 50% are indicated at the branch nodes. GenBank accession numbers and their geographic isolation sources of reference sequences are shown in parentheses. The scale bar represents 3% dissimilarities among different *Mycena* sp.

Three *Mycena* OTUs, designated OTU117, OTU148 and OTU155, were obtained in this study. Phylogenetically, all were combined into one clade with the two species *M. epipterygia* and *M. alexandri*. The strain BZZ was positioned in a different clade (Fig. 4). These three OTUs were distributed differently among the growth phases of *G. elata*. Phase M contained all three OTUs, but P only contained OTU155, while B had none. The surrounding soils contained only OTU117. The percentage of OTU155 was relatively higher than that of OTU117 and OTU148. However, in general, *Mycena* occupied a very low percentage of the total reads (an average of less than 1.0%).

## Discussion

Currently, our knowledge of the fungal biology of *G. elata* is very limited, although this species is widely distributed and has high medicinal value [﻿23﻿, ﻿25﻿, 26]. This study is the first to use culture-independent molecular techniques to characterize the fungal community associated with *G. elata*. As previously reported for most other orchids [18], the broader range of fungi than previously assumed, involving many fungal families from the phyla Basidiomycota and Ascomycota, may be related to the germination and vegetation growth of *G. elata* (Fig. 1).

The most important finding in this study is that the fungal communities associated with *G. elata* changed with the developmental phases (Figs. 1, 2). Generally, seedling-mycobiont specificity appears to be narrower than in adult plants [21, 22]. However, fungal diversities declined with development phases of *G. elata* in this case (also see Additional file 4: Figure S1). For Basidiomycota, no OTUs with known names overlapped between the soils and *G. elata* tubers, with the exception of several rare unidentified OTUs (Fig. 1b). With the developmental phases of *G. elata*, the fungal species richness declined but the relative abundance of predominate OTUs increased, from diverse basidiomycetes (11 genera, with a percentage of 20%) in phase P to a single Agaricales OTU1 in phase B (with a percentage of 98%). In phase P, the shared genera in three replicates included *Hyphodontia* (Trechisporales), *Sistotrema* (Cantharellales), *Russula* (Russulales), and *Mingxiaea* (Tremellales). *Hyphodontia*, *Sistotrema* and *Russula* are common in the fungal communities associated with orchids, e.g., *Erythrorchis altissima* [37], *Corallorhiza* [38], and *Limodorum* [18, 39]. There is no report of *Mingxiaea* as a mycorrhizal fungus of orchids. The genus *Resinicium* is common in *Gastrodia* species, e.g., *G. similis* [32] and *G. sesamoides* [33]. In the present study, one species, *Resinicium bicolor,* was numerically dominant in phase M, but it was rarely observed in phase P and the surrounding soils and was completely absent in phase B. These OTUs were rare in soils. Interestingly, fungal species had limited overlap between soil and the tubers of *G. elata* (Figs. 1, 2). In soils, the dominant fungi, including *Simocybe*, *Psathyrella* and *Conocybe*, were absent in the *G. elata* tubers, regardless of the growth phase (Fig. 1). Among these genera, only *Psathyrella* was previously observed in the orchid *Neottia ovata* [40]. Similarly, the dominant fungi in the *G. elata* tubers, e.g., *Resinicium*, *Mariannaea*, *Trichoderma* and *Clonostachys,* were rare fungi in the soil samples. *Hyphodontia* and *Sistotrema,* which were the dominant fungi in group P, were absent in the soil samples (Fig. 1). However, it was possible to detect these fungi with increased sequencing depth. Our data indicated that the fungal colonization in *G. elata* was highly dynamic, and *G. elata* was likely to select different basidiomycete fungi throughout the growth process.

Diverse ascomycetes, including *Clonostachys*, *Trichoderma*, *Mariannaea*, *Cladophialophora*, *Lecanicillium*, *Chaetosphaeria*, *Leptodontidium*, and *Fusarium*, were observed in *G. elata* as well as in the soils (Fig. 1c). Most of these genera are common nonmycorrhizal endophytes associated with orchids [37, 41]. *Clonostachys rosea* and *Trichoderma chlorosporum* acted as potential biocontrol agents and promoted the growth of the orchid *Dendrobium nobile* [41]. *Trichoderma harzianum* was proven to significantly promote the seedling survival and growth of the orchid *Guarianthe skinneri* [42]. *Fusarium,* as an endophytic fungus, has been found to play an important role in the growth and survival of *D. friedericksianum* [43]. However, there is controversy about the role of these nonmycorrhizal endophytes in orchids. It is possible that surface contaminants could be mistakenly identified as orchid nonmycorrhizal endophytes [44]. Because no certain role of ascomycetes associated with *G. elata* is reported, one should be cautious about their role in the growth of *G.elata*.

There is conflict regarding the fungal specificity of *Gastrodia* species. Some authors think that *Gastrodia* species have a strong preference to associate with *Mycena* fungi, e.g., *Mycena* cf. *quiniaultensis* and *Mycena chlorophos* in *G. flavilabela* [24] and several *Mycena* species in *G. confusa* [31]. Since the fungus *M. osmundicola* has been isolated and identified [28, 30], no novel fungi beyond the *Mycena* species have been reported to associate with *G. elata* in the wild until now, which misread that *G. elata* likely has a strong specificity for the fungal family Mycenaceae at the early stage of growth. However, in our study, the proportion of *Mycena* was very low across the different growth phases of *G. elata* as well as in the surrounding soils (also see Additional file 6: Table S5 for the distribution of *Mycena* spp. in each sample). Diverse *Mycena* species have been previously proven in vitro to promote the seed germination of *G. elata*; however, these fungi were isolated from other orchids [36, 45]. In addition, several studies have shown that another fungal species in six genera isolated from different species of orchids are also capable of promoting the seed germination of *G. elata* [46]. Therefore, our data indicated that the fungi involved in the germination of *G. elata* could come from outside the family Mycenaceae. Interestingly, our data indicated that three OTUs previously reported in Italy were grouped with *M. epipterygia* [47] and *M. alexandri* in Sweden (Fig. 4). In addition, no OTUs from *Mycena* in this study were closely related to *M. osmundicola*, a fungus widely applied in the artificial cultivation of *G. elata* [28, 30]. *Mycena* strain BZZ, which is currently used in the cultivation of *G. elata* in Zhaotong, was closely related to a strain *Mycena* sp*.* (Fig. 4), which was isolated from *G. nipponica* in Japan [34]. These data suggest that *Mycena* strains being able to promote the germination of *G. elata* may be distributed globally.

Traditionally, *G. elata* is considered to switch from a specific single-fungus relationship (*Mycena*) to another single-fungus relationship (*Armellaria*) during its life cycle [30, 48]. *Mycena* must be replaced by *A. mellea* late in protocorm development [28], assuming that this replacement provides the *G. elata* with access to the greater reserves of organic carbon contained in the massive woody substrates exploited by *A. mellea* [49, 50]. Our molecular data suggested that the switch was more complex than previously assumed. Because diverse fungi are associated with different growth phases of *G. elata* (Figs. 1, 2; also see the discussion above), multiple fungal switches may occur at different development phases after germination, e.g., from *Resinicium* in phase M to unknown Agaricales OTU1 in phase B. There is evidence that diverse litter- or wood-decaying fungi, such as *Resinicium*, *Campanella* and *Marasmius* species, can provide carbon and nutrients to *Gastrodia* species [32, 33]. Therefore, our data indicated that at least two different fungi other than *Mycena* species might be involved in development before the arrival of *Armillaria* to sustain the growth of *G. elata* into mature tubers. To achieve this, these two fungi need to be cultured and reinoculated successfully, as previously indicated [18]. Unfortunately, our multiple efforts to culture these two fungi failed.

Wild orchids have a broad range of germination stages characterized by a high degree of variability in either the timing of seed germination or the rate of seed development. This variability could have primarily resulted from the absence of mycelia of appropriate fungi at the seed microsite [3]. Therefore, the presence of specific mycorrhizal fungi contributes, at least partly, to the spatial distribution and coexistence of orchid species [51]. Recent studies suggest that mycorrhizal fungi have an aggregated distribution within the habitats of orchids [52]. Higher abundances of fungal symbionts are typically found close to adult plants [3, 53]. In a study of the fungi *Tulasnella* spp. associated with *G. pubescens*, McCormick et al. (2016) found that established plants of *G. pubescens* that are < 50 cm apart associate with a single abundant fungal genet, while those > 50 cm apart associate with multiple fungal genets [54]. In addition to the fungal community associated with the development of *G. elata*, we originally tried to design our experiments to assess the spatial variation of the fungi. However, it is difficult to contain all the growth phases of *G. elata* in a single hole (microsite). After nearly one year of cultivation, some holes had no germination (empty holes) and some halted at protocorms with no further development into immature tubers (e.g., phases M and B). Therefore, we had to mix tubers at the same growth phase from different holes (also see Additional file 7: Table S6 The tubers at different growth phases in five holes). These data indirectly indicated that the distribution of fungi associated with *G. elata* in this site was highly heterogeneous; therefore, it was worth detailing the fungal species inhabiting soils and plant litters in these microsites. Finally, no *A. mellae* were detected in any samples in this study, and the possible reason was the absence or very low occurrence of this fungus in this site. Although our report revealed the novelty in fungal communities associated with *G. elata* during the developmental phases, only few holes were successfully germinated in one geographic site, which undoubtedly caused a degree of fungal community bias. In the future, more research is needed to determine whether *Resinicium* and OTU1 are common fungal switches during different development phases and in different habitats of *G. elata* and at what time point following these switches *Armillaria* finally arrives to complete the life cycle of *G. elata*.

## Conclusions

*Gastrodia elata* is an important herbal medicine in Asian countries. However, there has been no detailed study on the fungal community associated with the growth phases of *G. elata* in the wild since 1989. In the present study, *G. elata* in Zhaotong, a core production area in China, was selected, and high-throughput sequencing was used to characterize its fungal community. Tubers with different growth phases, along with their surrounding soils, were collected and sequenced*.* The data revealed that *G. elata* was associated with a broad range of fungi beyond *Mycena* species. These fungi changed with the growth phases of *G. elata*. In addition, this study suggested a complex switch of the fungal community with the growth of *G. elata*. The *Mycena* spp. were clearly not the majority of fungi associated with the germination and early growth of *G. elata*. This study is the first report on the fungal dynamics of *G. elata* and will provide key insight into the *G. elata* –mycorrhizal fungal interaction in the future.

## Methods

### Description of the sampling site

**Fig. 5:**
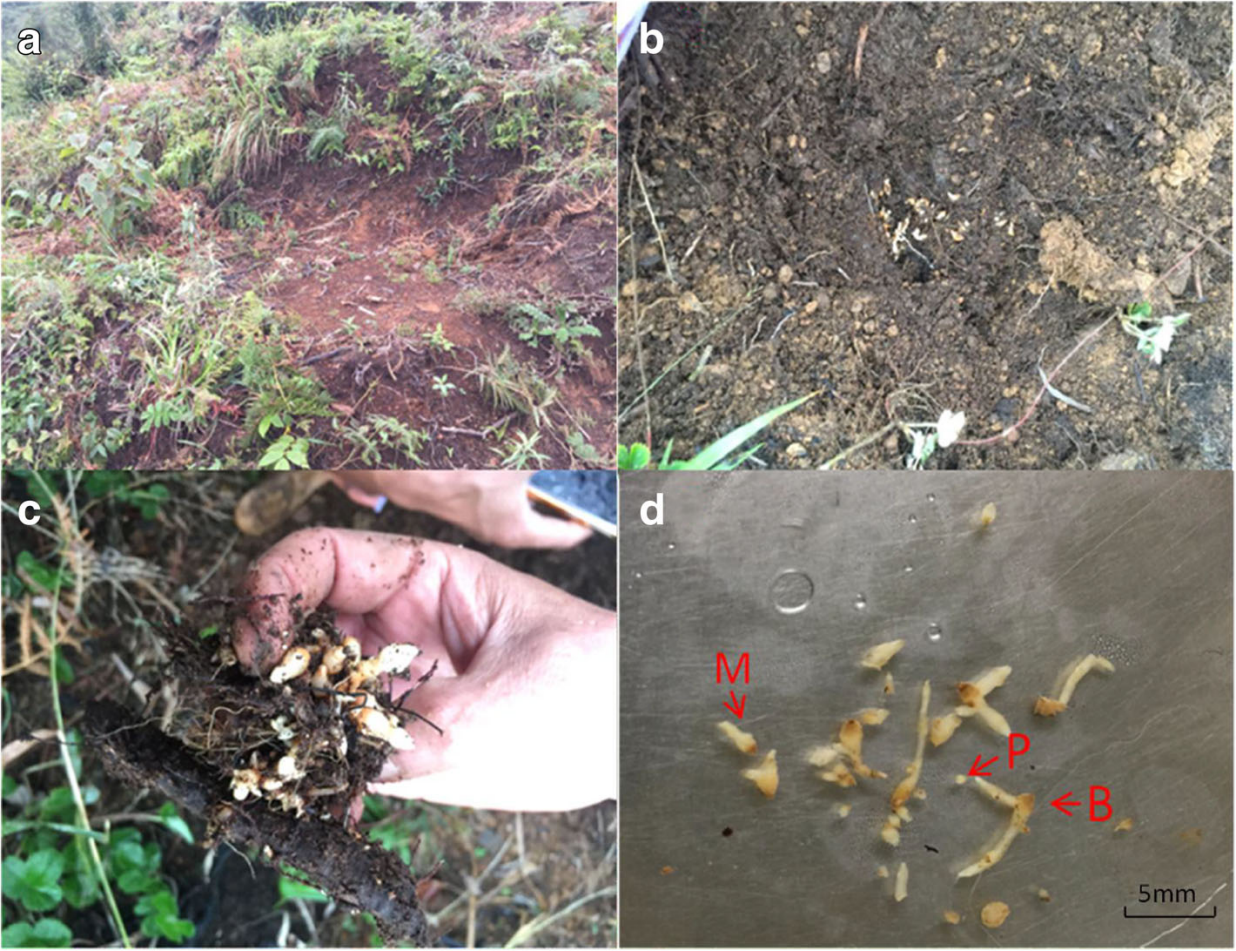
Sampling sites of *G. elata*. Planting holes of *G. elata* in the wild (**a**); the uncovered planting holes with *G. elata* tubers (**b** and **c**); the different growth phases of *G. elata* (**d**). P: protocorm; M: rice-like *G. elata*; B: propagation vegetation tubers of *G. elata*

Zhaotong is located in the northeast of Yunnan Province, China, and is well known for its high-quality *G. elata* production [55]. Our study site was thus selected in the core producing area of *G. elata*, Xiaocaoba, Zhaotong (27°48′7.99 N, 104°15′57.96E). This site grows sporadic trees and shrubs, including *Sorbus pohuashanensis*, *Castanopsis delavayi*, *Dipentodon sinicus*, *Corylus chinensis*, *Phoebe zhennan*, *Cerasus serrula*, *Acer oliverianum*, *Alnus cremastogyne*, and *Cunninghamia* sp. Traditionally, wild *G. elata*was distributed in this area. To bait mycorrhizal fungi, 10 planting holes (80 cm (L) × 50 cm (W) × 20 cm (H)) were randomly dug in nearly 100 m^2^ of the area in July 2016. The litter of the trees (primarily Fagaceae tree leaves, which are traditionally thought to be good sources for mycorrhizal fungi growth on *G. elata* [48]) were collected and laid on the bottom of holes. Seeds of *G. elata* (from 8 to 10 capsules per hole) were well distributed onto plant materials and covered with a 5–10 cm layer of soil. In August 2017, to obtain *G. elata* tubers at different development phases, these holes were uncovered and *G. elata* tubers were collected (Fig. 5). Unfortunately, a half of holes failed to germinate. Among the holes with tubers, most of them failed to contain all the growth phases of *G. elata* in a single hole (see Additional file 7: Table S6 The tubers at different growth phases in five holes). We collected all tubers and categorized these tubers according to their size into three groups representing different growth phases of *G. elata*, including protocorms (phase P, size 0.5–1 mm), rice-like tubers (phase M, size 3-5 mm), and propagation vegetation tubers (phase B, size > 8 mm). The different phases of *G. elata* tubers from different holes were evenly mixed in equal proportions and divided into three replicates, which contained dozens of tuber individuals without damage in order to avoid the cross contamination of endophytic fungi. The *G. elata* tubers were washed with a large amount of sterile water after two minutes of surface disinfection with 0.5% sodium hypochlorite and crushed, then immediately stored at − 80 °C for subsequent analysis. To verify whether there is an overlap of fungal community between *G. elata* tubers and the surrounding soils, we collected 500 g of soil from each hole and a total of 2500 g of soil was obtained from 5 holes. Then, we repeatedly mixed the soils using method of coning and quartering until they were well mixed, after which we randomly took three subsamples of soils as methodological replicates to increase the recovery of fungi from the pooled samples. Soils were immediately stored at − 80 °C for subsequent analysis. In this study, a total of 12 samples, including three 250 mg samples of tubers from each growth phase and three 250 mg samples of soils, were used for DNA extraction.

### DNA extraction and library construction

The genomic DNA of the plant tubers was extracted using CTAB [56]. Genomic DNA from the surrounding soils was extracted using a commercial DNA extraction kit (PowerSoil® DNA Isolation Kit, USA). The DNA quality was monitored using 0.8% agarose gel electrophoresis, and the extracted DNA was diluted to a concentration of 1 ng/μL and stored at − 20 °C until further processing. The diluted DNA was used as a template for the PCR amplification of fungal ITS genes with the barcoded primers and a HiFi Hot Start Ready Mix (KAPA, USA).

The ITS region of the fungi was amplified using the primers 1743F (5′-CTTGGTCATTTAGAGGAAGTAA-3′) and 2043R (5′-GCTGCGTTCTTCATCGATGC-3′) [57]. The 5′-barcoded amplicons were generated by PCR under conditions of 2 min at 95 °C, followed by 35 cycles at 95 °C for 30 s, annealing at 55 °C for 1 min, extension at 72 °C for 1 min, and a final extension at 72 °C for 10 min. Amplicon quality was visualized using gel electrophoresis, purified with AMPure XP beads (Agencourt) and amplified for another round of PCR. After purification with the AMPure XP beads again, the final amplicon was quantified using a Qubit dsDNA assay kit. Equal amounts of purified amplicon were pooled for subsequent sequencing at the Shanghai OE Biotech. Co., Ltd. (Shanghai,China).

### Data analysis

Raw sequencing data were in the FASTQ format. Paired-end reads were preprocessed using Trimmomatic software [58] to detect and remove ambiguous bases (N). We also removed low-quality sequences with average quality score below 20 using the sliding window trimming approach. After trimming, paired-end reads were assembled using FLASH software [59]. The parameters of assembly were 10 bp of minimal overlapping, 200 bp of maximum overlapping and a 20% maximum mismatch rate. The sequences were further denoised as follows: reads with ambiguous, homologous sequences or below 200 bp were abandoned. Reads with 75% of bases above Q20 were retained. Reads with chimeras were detected and removed. These steps were performed using QIIME software (version 1.8.0) [60].

Clean reads were subjected to primer sequence removal and clustering to generate OTUs using UPARSE software with a 97% similarity cutoff [61]. The representative read of each OTU was selected using the QIIME package. All representative reads were annotated and blasted against the Unite database using BLAST [62]. At the phylum level, nonfungal reads and unidentified reads were merged as “Other” for further analysis.

Species accumulation curves [63] were analyzed using QIIME. All the resulting curves tended to approach the saturation plateau, indicating that the number of sequenced reads in each sample was reasonable (see Additional file 4: Figure S1 for species accumulation curves). The probability of new species detected declined significantly at ~ 5000 reads per sample with the exception of the soil. To compare the fungal diversity in tubers across all growth phases, we had to sacrifice some species to ensure that all the samples had the same sequencing depth because one sample of *G. elata* (P1) had the lowest reads of 4531 (an additional table of the description of sample tags also shows this in detail [see Additional file 1: Table S1]). Fortunately, the numbers of OTUs for *G. elata* had not significantly declined at the depth of 4530 tags. The sequences were randomly subsampled to avoid differences based on sequencing effort, leaving 4530 reads for further analysis as previously described [64, 65]. Three subsamples of soils as methodological replicates were thus pooled into one sample, and only mean values were compared in further analyses.

Venn diagram was used to measure the overlap between four fungal communities and was conducted using the “VennDiagram” package in R software. Heatmapping of the top 10 OTUs used for fungi distribution in different groups was performed using the R package “pheatmap.” Other histogram and line charts were plotted using Excel 2007 and GraphPad Prism 7 (GraphPad Software, Inc., CA, USA).

### Phylogenetic analyses

Because *Mycena* has been widely proven to germinate *G. elata* [35, 36, 48], the ITS sequences of 20 *Mycena* references were downloaded from the NCBI database (sequence information of *Mycena* spp. is shown in Additional file 8: Table S7). Combined with our three *Mycena* sequences obtained from high-throughput sequencing and a strain of *Mycena* sp. (BZZ) previously isolated from *G. elata* protocorms in the Zhaotong area, a total of 24 partial ITS sequences were used for phylogenetic analyses. Bayesian (BI) analyses were performed using MrBayes v. 3.2.1 [66] based on the models selected by jModeltest v.2.1.4.The analyses lasted until the average standard deviation of split frequencies was below 0.01 with trees saved every 1000 generations. The first 25% of the trees were removed as the burn-in phase, and the remaining trees were used to calculate posterior probabilities. Posterior probability values of the BI analyses (BPP) over 95.0% were considered significant.

## Additional files

Additional file 1: **Table S1.** Description of sample Tags. (DOCX 12 kb)

Additional file 2: **Table S2.** OTUs table. (XLSX 74 kb)

Additional file 3: **Table S3.** OTUs table after subsampled. (XLSX 61 kb)

Additional file 4: **Figure S1.** Species accumulation curves. (TIF 2414 kb)

Additional file 5: **Table S4.** Venn diagram for OTUs distribution. (XLSX 18 kb)

Additional file 6: **Table S5.** Distribution of *Mycena* sp. in each sample. (DOCX 13 kb)

Additional file 7: **Table S6.** The tubers at different growth phases in five holes. (DOCX 13 kb)

Additional file 8: **Table S7.** Sequence information of *Mycena* spp. (DOCX 15 kb)

## Data Availability

All data analyzed during this study are included in this published article and its supplementary information files. Additional raw data from sequencing and quality control datasets are available from the corresponding author upon reasonable request.

## Abbreviations

BLAST: Basic local alignment search tool
ITS: Internal transcribed spacer
OTU: Operational taxonomic units
