# Supplementary material for: Dynamics of fungal communities during Gastrodia elata growth
Source: BMC Microbiol. 2019 Jul 10;19:158. doi: 10.1186/s12866-019-1501-z (PMC6617676; doi:10.1186/s12866-019-1501-z)
Supplement: Supplementary file 1 — Table S1. Description of sample Tags. (DOCX 12 kb) [file 12866_2019_1501_MOESM1_ESM.docx]

**Additional file 1**

**sTab.1 Description of sample Tags**

| Sample ID | Valid tags | % of high-quality reads in tuber; soil | | OTU counts | Subseq in depth  4530 OTU counts | |
| --- | --- | --- | --- | --- | --- | --- |
| P1 | 4531 | 97.90% | 103 | | 103 |  |
| P2 | 30656 | 98.17% | 133 | | 111 |  |
| P3 | 13611 | 97.90% | 88 | | 77 |  |
| M1 | 38636 | 97.96% | 114 | | 63 |  |
| M2 | 39589 | 98.48% | 95 | | 53 |  |
| M3 | 39754 | 98.52% | 103 | | 62 |  |
| B1 | 39420 | 98.26% | 50 | | 30 |  |
| B2 | 37680 | 98.07% | 30 | | 14 |  |
| B3 | 41185 | 98.34% | 39 | | 16 |  |
| S1 | 39889 | 95.01% | 569 | | 360 |  |
| S2 | 38407 | 94.93% | 597 | | 372 |  |
| S3 | 38759 | 94.64% | 595 | | 373 |  |
