# Supplementary figures and images for: Dynamics of fungal communities during Gastrodia elata growth
Source: BMC Microbiol. 2019 Jul 10;19:158. doi: 10.1186/s12866-019-1501-z (PMC6617676; doi:10.1186/s12866-019-1501-z)

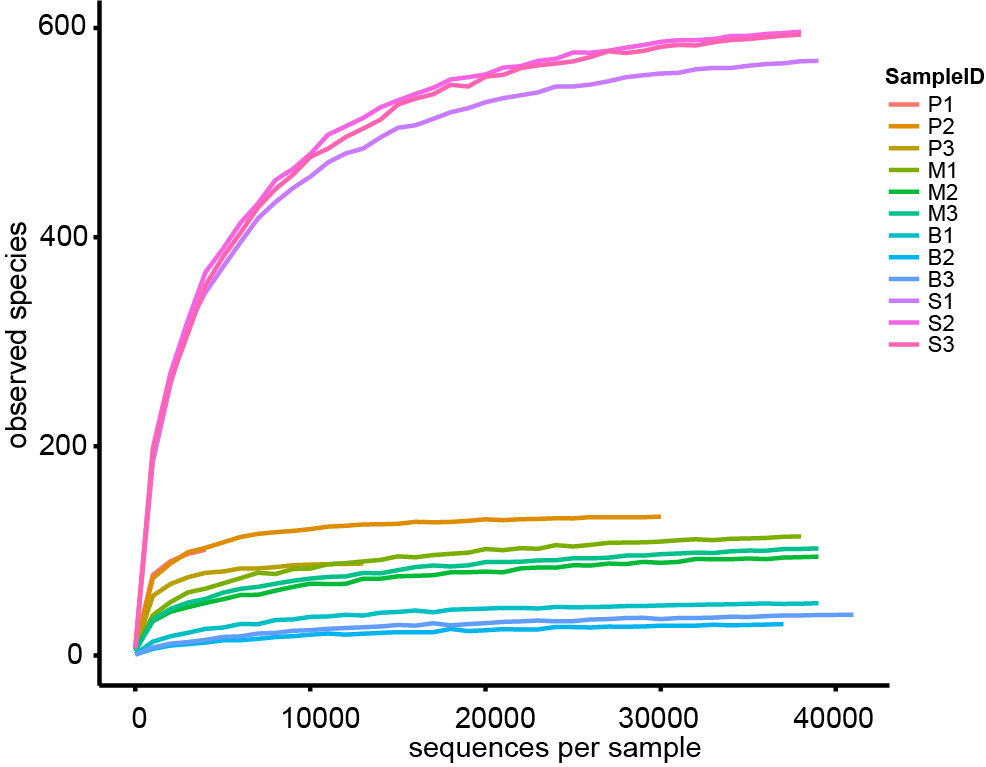

Supplement: Supplementary file 4 — Figure S1. Species accumulation curves. (TIF 2414 kb) [file 12866_2019_1501_MOESM4_ESM.tif]
