# Supplementary material for: Dynamics of fungal communities during Gastrodia elata growth
Source: BMC Microbiol. 2019 Jul 10;19:158. doi: 10.1186/s12866-019-1501-z (PMC6617676; doi:10.1186/s12866-019-1501-z)
Supplement: Supplementary file 6 — Table S5. Distribution of Mycena sp. in each sample. (DOCX 13 kb) [file 12866_2019_1501_MOESM6_ESM.docx]

**Additional file 6**

**sTab. 5 Distribution of *Mycena* sp. in each sample**

|  | P(%) | M(%) | B(%) | S(%) |
| --- | --- | --- | --- | --- |
| OTU117 | 0.000±0.000 | 0.021±0.019 | 0.000±0.000 | 0.005 |
| OTU148 | 0.000±0.000 | 0.008±0.002 | 0.000±0.000 | 0.000 |
| OTU155 | 1.524±1.437 | 0.027±0.032 | 0.000±0.000 | 0.000 |
| Mycena average | 0.508±1.047 | 0.019±0.021 | 0.000±0.000 | 0.002 |

The numbers in table represents the relative abundance of each OTU. P: protocorm M: rice-like *G. elata*, B: propagation vegetation tubers S: surrounding soil group
