# Supplementary material for: Dynamics of fungal communities during Gastrodia elata growth
Source: BMC Microbiol. 2019 Jul 10;19:158. doi: 10.1186/s12866-019-1501-z (PMC6617676; doi:10.1186/s12866-019-1501-z)
Supplement: Supplementary file 7 — Table S6. The tubers at different growth phases in five holes. (DOCX 13 kb) [file 12866_2019_1501_MOESM7_ESM.docx]

**Additional file 7**

**sTable 6 The tubers at different growth phases in five holes**

| **Growth phases** | Hole 1 | Hole 2 | Hole 3 | Hole 4 | Hole 5 |
| --- | --- | --- | --- | --- | --- |
| Protocorms | × | √ | √ | × | √ |
| Rice-like tubers | √ | × | √ | √ | √ |
| Propagation vegetation tubers | √ | × | × | √ | √ |

√, present; ×, absence.
