# Supplementary material for: Dynamics of fungal communities during Gastrodia elata growth
Source: BMC Microbiol. 2019 Jul 10;19:158. doi: 10.1186/s12866-019-1501-z (PMC6617676; doi:10.1186/s12866-019-1501-z)
Supplement: Supplementary file 8 — Table S7. Sequence information of Mycena spp. (DOCX 15 kb) [file 12866_2019_1501_MOESM8_ESM.docx]

**Additional file 8**

**sTable 7 Sequence information of *Mycena* spp.**

| Sample ID | GenBank Accession | length(bp) | Taxonomy | Source | Reference |
| --- | --- | --- | --- | --- | --- |
| M8 | JF908487.1 | 824 | *Mycena rosella* | Italy | (Osmundson et al., 2013) |
| M11 | JF908484.1 | 711 | *Mycena galopus* | Italy | (Osmundson et al., 2013) |
| M15 | JF908441.1 | 910 | *Mycena galericulata* | Italy | (Osmundson et al., 2013) |
| M20 | JF908468.1 | 605 | *Mycena epipterygia* | Italy | (Osmundson et al., 2013) |
| M22 | JF908415.1 | 736 | *Mycena citrinomarginata* | Italy | (Osmundson et al., 2013) |
| M30 | JF908492.1 | 750 | *Mycena crocata* | Italy | (Osmundson et al., 2013) |
| M31 | JF908430.1 | 732 | *Mycena rubromarginata* | Austria | (Osmundson et al., 2013) |
| M36 | JF908370.1 | 788 | *Mycena sanguinolenta* | Spain | (Osmundson et al., 2013) |
| M40 | JF908417.1 | 822 | *Mycena diosma* | Italy | (Osmundson et al., 2013) |
| M44 | JF908380.1 | 687 | *Mycena pelianthina* | Italy | (Osmundson et al., 2013) |
| M49 | KF537252.1 | 759 | *Mycena seminau* | Malaysia | (Chew et al., 2014) |
| M50 | KF537249.1 | 764 | *Mycena sinar* | Malaysia | (Chew et al., 2014) |
| M51 | KF537248.1 | 745 | *Mycena cahaya* | Malaysia | (Chew et al., 2014) |
| M52 | LC013373.1 | 722 | *Mycena sp.* | *Gastrodia nipponica*/Japan | (Kinoshita et al., 2016) |
| M1 | LC274964.1 | 673 | *Mycena chlorophos* | Japan | — |
| M25 | JN182201.1 | 628 | *Mycena pearsoniana* | USA | — |
| M3 | KC581347.1 | 1209 | *Mycena pura* | Canada | — |
| M46 | KT900146.1 | 1101 | *Mycena cinerella* | Norway | — |
| M47 | KT900145.1 | 1138 | *Mycena alexandri* | Sweden | — |
| M48 | KT900141.1 | 846 | *Mycena adscendens* | Sweden | — |
